# Insights into the Indian Peanut Genotypes for *ahFAD2* Gene Polymorphism Regulating Its Oleic and Linoleic Acid Fluxes

**DOI:** 10.3389/fpls.2016.01271

**Published:** 2016-08-25

**Authors:** Bhagwat Nawade, Tejas C. Bosamia, Radhakrishnan Thankappan, Arulthambi L. Rathnakumar, Abhay Kumar, Jentilal R. Dobaria, Rahul Kundu, Gyan P. Mishra

**Affiliations:** ^1^Department of Biotechnology, Directorate of Groundnut ResearchJunagadh, India; ^2^Department of Biosciences, Saurashtra UniversityRajkot, India; ^3^Department of Biotechnology, Indian Institute of Vegetable ResearchVaranasi, India

**Keywords:** *FAD2* gene, allele-specific PCR, fatty-acids, groundnut, O/L ratio, single nucleotide polymorphism

## Abstract

In peanut (*Arachis hypogaea* L.), the customization of fatty acid profile is an evolving area to fulfill the nutritional needs in the modern market. A total of 174 peanut genotypes, including 167 Indian cultivars, 6 advanced breeding lines and “SunOleic95R”—a double mutant line, were investigated using AS-PCRs, CAPS and gene sequencing for the *ahFAD2* allele polymorphism, along with its fatty acid compositions. Of these, 80 genotypes were found having substitution (448G>A) mutation only in *ahFAD2A* gene, while none recorded 1-bp insertion (441_442insA) mutation in *ahFAD2B* gene. Moreover, 22 wild peanut accessions found lacking both the mutations. Among botanical types, the *ahFAD2A* mutation was more frequent in ssp. *hypogaea* (89%) than in ssp. *fastigiata* (17%). This single allele mutation, found affecting not only oleic to linoleic acid fluxes, but also the composition of other fatty acids in the genotypes studied. Repeated use of a few selected genotypes in the Indian varietal development programs were also eminently reflected in its *ahFAD2* allele polymorphism. Absence of known mutations in the wild-relatives indicated the possible origin of these mutations, after the allotetraploidization of cultivated peanut. The SNP analysis of both *ahFAD2A* and *ahFAD2B* genes, revealed haplotype diversity of 1.05% and 0.95%, while K_a_/K_s_ ratio of 0.36 and 0.39, respectively, indicating strong purifying selection pressure on these genes. Cluster analysis, using *ahFAD2* gene SNPs, showed presence of both mutant and non-mutant genotypes in the same cluster, which might be due the presence of *ahFAD2* gene families. This investigation provided insights into the large number of Indian peanut genotypes, covering various aspects related to O/L flux regulation and *ahFAD2* gene polymorphism.

## Introduction

Cultivated peanut or groundnut (*Arachis hypogaea* L.) is an allotetraploid crop (2*n* = 4*x* = 40, AABB) having two subspecies, spp. *hypogaea* and spp. *fastigiata* (Krapovickas and Rigoni, 1960). It is cultivated in more than 100 countries, mostly by the small and marginal farmers, under low-input conditions (Bhauso et al., 2014; Sarkar et al., 2014). Peanut is one among the major oilseed crops, which contribute to the bulk of total oil production worldwide (Mishra et al., 2015). Indian vegetable oil economy is world's fourth largest after USA, China and Brazil. India ranks first in acreage, occupying 5.25 M ha under cultivation and second in production (9.47 M tons) in the world, after China (16.91 M tons) (FAOSTAT, 2014).

In peanut seed oil, two unsaturated fatty acids (UFA), oleic acid (C18:1, Δ9), a mono UFA (MUFA) and linoleic acid (C18:2, Δ9, Δ12), a poly UFA (PUFA) contribute around 80% of the total oil composition. Further, a saturated fatty acid (SFA), palmitic acid contributing to about 10%, whereas, rest 10% is constituted of up to 9 other fatty acids (Janila et al., 2016). Thus, the flavor, shelf-life, and nutritional quality of peanut seeds and its products are reliant on the proportion of three main fatty acids *viz*., oleic, linoleic and palmitic acid present in its oil (Derbyshire, 2014). Oils containing high percentage of linoleic acid are prone to oxidation, leading to rancidity, off-flavors, and short shelf-life (Mondal et al., 2010). Oleic acid has 10-fold higher auto-oxidative stability than linoleic acid; therefore, high O/L peanut has a longer shelf life (O'Keefe et al., 1993). The palmitic acid is reported to increase the risk for multiple life-threatening diseases such as cardio-vascular diseases (CVD) and atrial-fibrillation (Wang et al., 2015a).

High-oleic oil is an excellent solution for food manufacturers, looking for healthy alternatives to saturated or hydrogenated oils (Cao et al., 2013). Its neutral flavor and odor, make the oil highly suitable for a wide range of food related applications, including spray coating and frying (Pandey et al., 2014). A diet with a high oleic acid and low palmitic acid, is an exceptional way to reduce the risk of heart diseases, promotes a healthier ratio of high density lipoprotein (HDL) to low density lipoprotein (LDL), and reduces triacylglycerol and blood glucose levels (Vassiliou et al., 2009; Janila et al., 2013).

Normal peanut genotypes contain about 36–70% oleic while 15–43% linoleic acid (Knauft et al., 1993). However, in 1987, Norden and co-workers identified the first high-oleate mutant lines, F435 with about 80% oleic acid and 2% linoleic acid. So far, more than 50 high-oleate peanut cultivars were registered worldwide, which are derived through traditional breeding, mutagenesis, marker-assisted selection (MAS) and marker assisted backcross breeding (MABB) (Wang et al., 2015c; Janila et al., 2016). Following conventional breeding methods, the first high oleate peanut line, “SunOleic95R” was bred in USA (Gorbet and Knauft, 1997); whereas, “Tifguard High O/L” was developed using MAS (Chu et al., 2011).

In plants, oleate to linoleate conversion is catalyzed by oleoyl-PC (phosphatidyl choline) desaturase, which exists in endoplasmic reticula and chloroplast membrane, and incorporates a second double bond to oleic acid (Schwartzbeck et al., 2001). In peanuts, two homeologous genes, *ahFAD2A* and *ahFAD2B* having 99% sequence similarity, are reported to regulate the desaturase activity (Jung et al., 2000b; Lopez et al., 2000). A single base pair (bp) substitution (G:C/A:T) mutation at 448 bp position in *ahFAD2A* gene, results in a missense amino acid from aspartic acid to asparagine (D150N). While, 1-bp insertion (A:T) mutation in *ahFAD2B* gene, at 442 bp position results in frame-shift mutation, which generates a premature stop codon (Jung et al., 2000b; Lopez et al., 2000). Both these mutations lead to the altered *ahFAD2* gene expression, resulting in reduced enzymatic activity, which leads to high O/L ratio in the mutant genotypes (Jung et al., 2000a; Chu et al., 2009). Recently, Wang et al. (2015b) has identified two natural mutant lines (PI342664 and PI342666) with 80% oleic acid, having substitutions of G448A in *FAD2A* (same as previously identified) and C301G in *FAD2B* (new mutation) for both mutant lines, resulting in a missense amino acid substitution of D150N, and H101D, respectively. However, many reports also indicate the epistatic regulation, presence of *ahFAD2* gene families, and existence of other unidentified genetic factors, conditioning fatty-acid content in peanut (Fang et al., 2012; Wang et al., 2015b,d). The various QTLs for *ahFAD2* genes were reported to be located on 6th and 9th linkage group of both A and B genomes of cultivated peanut (Pandey et al., 2014; Wang et al., 2015a).

To enhance the efficiency of peanut breeding program for high oleate, different molecular tools for accurate genotyping of ah*FAD2* genes includes; cleaved amplified polymorphic sequences (CAPS) markers for *ahFAD2A* (Chu et al., 2007) and ah*FAD2B* alleles (Chu et al., 2009), real-time PCR (Barkley et al., 2010, 2011) and allele-specific PCR (AS-PCR) assays (Chen et al., 2010; Yu et al., 2013). These tools have been successfully utilized for the screening of peanut germplasm (Chu et al., 2007; Wang et al., 2011a,b, 2013b; Mukri et al., 2012). However, no such efforts have been reported for the Indian peanut cultivars and advanced breeding lines, which are being used in improving the O/L ratio of the Indian genotypes by the breeders.

Looking at the availability of various *ahFAD2* gene mutant high oleic lines in peanut, identification of Indian peanut cultivars for *ahFAD2* gene polymorphism and fatty acid composition seems essential for boosting the peanut breeding programme for oil quality traits. Therefore, the present study was aimed to find the relationship between *ahFAD2* allele polymorphism and its fatty-acid composition, especially O/L fluxes in Indian peanut genotypes.

## Materials and methods

### Plant materials

A total of 196 peanut genotypes; including 167 Indian cultivars, 6 advance breeding lines, SunOleic 95R− a high oleic (~80%) genotype, and 22 wild-species, representing different section were used for this study (Tables 1, 2). The seeds of these genotypes were obtained from the Genetic Resources Section of the ICAR-Directorate of Groundnut Research (DGR), Gujarat (India).

**Table 1:** **Fatty acid composition (%), O/L ratio and ***ahFAD2A*** substitution mutation in the 167 peanut varieties and six advance breeding lines**.

**S. no**.	**Genotypes**	**Market type**	**Palmitic acid (C16:0)**	**Stearic acid (C18:0)**	**Oleic acid (C18:1)**	**Linoleic acid (C18:2)**	**Arachidic acid (C20:0)**	**Gadoleic acid (C20:1)**	**Behenic acid (C22:0)**	**Lignoceric acid (C24:0)**	**O/L ratio**	**Substitution mutation in *ahFAD2A***
1	SunOleic95R	VR	6.93	2.28	80.18	7.34	0.78	1.26	1.00	0.23	10.92	+
2	RG510	VR	10.07	3.98	63.45	19.04	1.10	0.83	1.21	0.31	3.33	+
3	Kadiri71-1	VR	9.64	3.20	63.50	20.12	1.06	0.94	1.19	0.36	3.16	+
4	GG16	VR	9.00	2.25	63.96	20.58	0.93	1.01	1.75	0.52	3.11	+
5	UF70-103	VR	9.90	3.20	62.22	20.33	1.18	1.16	1.58	0.42	3.06	+
6	GG14	VR	10.65	3.17	59.50	21.17	1.29	1.10	2.21	0.88	2.81	+
7	GJG17	VR	9.77	2.30	61.35	22.05	1.03	1.08	1.87	0.54	2.78	+
8	GG13	VR	10.25	2.60	59.63	23.01	1.15	1.24	1.78	0.31	2.59	+
9	GG11	VR	10.46	2.52	58.86	22.73	1.21	1.23	2.28	0.54	2.59	+
10	GG 15	VR	10.43	2.32	59.95	23.32	0.95	1.02	1.60	0.41	2.57	+
11	Karad 4-11	VR	10.02	1.98	60.76	23.77	0.75	1.01	1.14	0.27	2.56	+
12	RS1	VR	10.43	2.13	59.97	23.57	1.04	0.89	1.30	0.41	2.54	+
13	M13	VR	11.51	2.86	57.81	24.10	1.04	1.04	1.22	0.22	2.40	+
14	M548	VR	11.03	2.83	57.08	24.61	1.18	0.91	1.78	0.55	2.32	+
15	S230	VR	10.19	3.04	58.33	25.18	0.99	0.66	1.12	0.23	2.32	+
16	Punjab1	VR	10.95	2.45	58.09	25.11	0.75	0.80	1.24	0.42	2.31	+
17	GAUG10	VR	11.15	2.63	57.47	24.94	1.12	0.92	1.38	0.39	2.30	+
18	M37	VR	10.77	2.54	57.80	25.50	0.92	0.98	1.16	0.25	2.27	+
19	Faizapur1-5	VR	10.68	2.56	57.21	25.36	1.00	1.06	1.66	0.49	2.26	+
20	DSG1	VR	10.79	2.48	56.14	26.00	1.13	1.18	1.72	0.53	2.16	+
21	TMV4	VR	10.70	2.17	55.86	25.98	1.09	1.24	2.13	0.82	2.15	+
22	GG12	VR	11.09	2.34	55.03	26.14	0.97	1.09	1.80	0.54	2.11	+
23	CSMG9510	VR	10.77	3.16	55.35	26.94	1.07	0.84	1.57	0.32	2.05	+
24	M335	VR	11.90	2.94	54.42	26.64	1.16	1.04	1.61	0.29	2.04	+
25	TMV1	VR	10.15	2.40	55.72	27.52	0.91	1.02	1.62	0.66	2.02	+
26	Chandra	VR	12.12	2.70	54.17	27.50	1.01	0.93	1.26	0.24	1.97	+
27	CSMG84-1	VR	12.79	4.08	50.48	25.91	1.76	1.05	3.30	0.52	1.95	+
28	TMV3	VR	10.50	2.13	54.71	28.58	0.95	1.12	1.54	0.48	1.91	+
29	T28	VR	10.68	1.65	53.99	28.59	1.68	1.61	1.38	0.42	1.89	+
30	Chitra / MA10	VR	12.94	3.63	50.55	28.67	1.31	1.00	1.53	0.28	1.76	+
31	GJG-HPS-1	VR	10.99	2.19	51.53	30.09	0.99	1.26	2.21	0.74	1.71	+
32	Somnath	VR	10.94	2.25	49.54	32.04	1.50	0.89	2.00	0.51	1.55	+
33	GJG22	VB	9.25	3.33	65.42	17.41	1.27	1.06	1.68	0.58	3.76	+
34	GG21	VB	9.51	3.68	64.78	17.86	1.24	0.82	1.70	0.43	3.63	+
35	GG20	VB	10.16	3.62	63.93	19.68	0.91	0.52	0.82	0.19	3.25	+
36	TMV10	VB	7.92	0.56	66.57	21.89	0.82	0.70	1.55	0.00	3.04	+
37	RG425	VB	10.00	3.54	61.52	21.11	0.95	0.87	1.65	0.36	2.91	+
38	BAU13	VB	9.63	2.90	63.30	21.98	0.69	0.30	1.00	0.20	2.88	+
39	ICGS76	VB	11.73	2.60	58.42	24.10	0.75	0.69	0.84	0.34	2.42	+
40	MA16	VB	11.02	2.58	57.65	24.33	1.08	0.86	1.81	0.60	2.37	+
41	HNG10	VB	8.97	1.98	59.81	25.53	0.96	0.82	1.41	0.53	2.34	+
42	ICGV86325	VB	9.93	2.04	59.37	25.52	0.87	0.87	0.67	0.58	2.33	+
43	Kadiri 7 Bold	VB	11.51	2.86	57.51	24.74	0.97	0.84	1.16	0.41	2.32	+
44	T64	VB	12.06	1.73	57.65	25.07	0.70	0.77	1.36	0.35	2.30	+
45	ALR1	VB	10.88	3.76	55.84	24.39	1.65	0.99	2.13	0.36	2.29	+
46	BG2	VB	10.98	1.89	58.47	26.00	0.47	0.98	0.98	0.23	2.25	+
47	M145	VB	10.75	2.26	57.63	25.81	0.92	0.88	1.27	0.49	2.23	+
48	ICGS5	VB	10.89	1.73	58.03	26.15	0.80	0.80	1.03	0.43	2.22	+
49	Kaushal	VB	11.07	2.69	56.63	26.25	1.02	1.03	1.00	0.23	2.16	+
50	CSMG884	VB	11.87	2.28	55.51	26.31	1.14	0.86	1.74	0.31	2.11	+
51	Kadiri2	VB	11.54	2.20	55.93	27.03	0.80	1.14	1.08	0.28	2.07	+
52	AK303	VB	12.29	1.68	55.86	27.56	0.70	0.88	0.75	0.28	2.03	+
53	BG3	VB	11.27	2.31	54.39	27.52	1.03	1.07	1.62	0.54	1.98	+
54	AK265	VB	11.19	3.15	54.38	27.68	1.09	0.68	1.37	0.46	1.96	+
55	RSB87	VB	11.79	1.87	54.16	27.87	0.88	1.19	1.61	0.21	1.94	+
56	TG1	VB	11.88	2.88	53.26	28.70	1.04	0.77	1.14	0.33	1.86	+
57	TGLPS3	VB	11.60	2.21	53.99	29.38	0.68	0.63	1.14	0.37	1.84	+
58	B95	VB	12.26	2.30	53.08	29.29	0.95	0.68	1.17	0.27	1.81	+
59	RS138	VB	11.41	2.38	52.38	29.22	1.14	0.87	2.17	0.43	1.79	+
60	M197	VB	12.80	3.50	51.20	29.06	1.15	0.80	1.31	0.19	1.76	+
61	HNG69	VB	11.41	2.11	51.90	29.56	1.04	1.18	2.13	0.67	1.76	+
62	Kadiri3	VB	11.78	1.70	49.52	28.97	1.21	0.92	1.60	4.30	1.71	−
63	DRG17	VB	12.53	3.10	49.24	30.94	1.19	0.90	1.61	0.48	1.59	+
64	Malika	VB	9.99	2.52	51.57	32.92	0.73	0.70	1.26	0.30	1.57	+
65	BG1	VB	13.32	2.46	48.47	32.23	0.89	0.71	1.34	0.35	1.50	−
66	Kadiri8 Bold	VB	11.38	2.67	47.25	34.21	1.07	1.03	1.85	0.55	1.38	+
67	Konkan Gaurao	VB	13.23	2.52	46.16	33.82	1.07	0.80	1.73	0.68	1.36	−
68	Girnar2	VB	13.23	3.40	44.94	34.63	1.12	0.69	1.52	0.47	1.30	−
69	M522	VB	14.05	3.06	44.99	34.98	0.91	0.54	1.00	0.20	1.29	−
70	BAU19^*^	VB	11.50	2.54	42.30	39.28	1.07	1.00	1.69	0.62	1.08	−
71	ICGV87846	VB	12.67	2.05	41.25	39.44	1.06	0.97	2.16	0.40	1.05	−
72	LGN2	VB	13.62	2.42	39.84	38.35	1.32	1.03	2.46	0.92	1.04	−
73	MH2	VAL	12.92	3.44	38.24	39.90	1.42	0.81	2.50	0.78	0.96	−
74	MH4	VAL	13.19	3.08	38.24	40.91	0.98	0.80	2.12	0.67	0.93	−
75	Gangapuri	VAL	12.32	2.83	41.60	39.22	1.13	0.74	1.73	0.45	1.06	−
76	Kopargaon3	VAL	11.77	3.26	44.79	35.97	1.22	1.10	1.53	0.36	1.25	−
77	TMV11	VAL	12.65	4.03	44.77	34.28	1.36	0.69	1.80	0.42	1.31	−
78	VRI5	SB	9.37	2.83	62.69	21.64	0.75	0.69	1.59	0.44	2.90	+
79	Tirupati3	SB	10.20	3.28	60.21	21.53	1.41	0.97	1.81	0.47	2.80	+
80	TPG41	SB	10.88	3.33	58.27	24.10	0.98	0.65	1.25	0.54	2.42	+
81	TAG24	SB	10.03	2.83	58.67	25.55	0.89	0.67	1.13	0.23	2.30	−
82	JL24	SB	11.75	1.93	57.88	25.26	0.74	0.78	1.22	0.42	2.29	−
83	GPBD4	SB	10.73	2.67	55.12	26.09	1.34	1.08	2.44	0.54	2.11	+
84	DH4-3^*^	SB	10.83	2.77	55.83	26.53	1.05	1.16	1.18	0.46	2.10	+
85	Chintamani1^*^	SB	10.51	1.79	56.24	27.03	0.94	1.06	1.67	0.76	2.08	+
86	Konkan Tapora^*^	SB	11.43	2.82	54.17	27.77	1.08	0.87	1.47	0.39	1.95	+
87	GG5	SB	12.79	4.08	50.48	25.91	1.76	1.05	3.30	0.52	1.95	−
88	R2001-3	SB	11.64	3.19	52.63	28.67	1.06	0.73	1.63	0.46	1.84	+
89	Kadiri9	SB	11.82	2.71	51.53	28.81	1.26	1.02	2.32	0.53	1.79	+
90	S206	SB	11.67	2.47	52.47	29.40	1.09	0.75	1.83	0.32	1.78	−
91	VG9816	SB	11.11	3.42	52.44	29.60	1.13	0.61	1.38	0.31	1.77	+
92	ALR2	SB	11.84	2.94	51.84	29.66	1.13	0.86	1.49	0.25	1.75	−
93	R2001-2	SB	12.23	2.91	51.69	29.73	1.00	0.67	1.39	0.39	1.74	+
94	TG17	SB	13.11	2.15	52.08	30.15	0.44	0.60	0.56	0.56	1.73	+
95	GJG9	SB	12.31	3.86	49.87	29.80	1.39	0.65	1.60	0.51	1.67	−
96	Girnar3	SB	12.55	2.75	49.72	32.27	0.73	0.53	0.85	0.61	1.54	−
97	TLG45	SB	11.84	2.91	48.47	33.02	1.06	0.70	1.56	0.45	1.47	+
98	Kadiri Harithandra	SB	11.99	3.05	48.33	33.06	1.03	0.73	1.39	0.42	1.46	−
99	TG26	SB	12.16	3.19	47.95	32.83	1.18	0.79	1.47	0.43	1.46	−
100	Jyothi	SB	13.22	4.88	45.05	31.30	1.75	0.73	2.69	0.38	1.44	−
101	TKG19 A	SB	10.96	2.01	49.40	34.43	1.13	0.70	1.00	0.38	1.43	+
102	TMV12	SB	13.91	3.54	46.15	32.54	1.74	0.38	0.65	1.09	1.42	−
103	VRI4	SB	13.64	2.53	46.93	33.11	1.05	0.81	1.49	0.45	1.42	−
104	GG4	SB	13.40	3.87	45.59	32.36	1.43	0.70	2.10	0.55	1.41	−
105	GG7	SB	13.40	3.87	45.59	32.36	1.43	0.70	2.01	0.55	1.41	−
106	J11	SB	12.42	4.70	44.11	31.78	1.33	0.81	3.71	1.15	1.39	−
107	ICG (FDRS 10)	SB	13.69	1.84	46.87	34.02	0.48	0.38	0.62	0.25	1.38	−
108	DRG12	SB	13.30	2.26	46.31	34.02	0.95	1.08	1.50	0.35	1.36	−
109	SG99	SB	12.76	2.46	46.52	34.32	1.01	0.79	1.66	0.48	1.36	−
110	DH40^*^	SB	12.68	3.86	45.35	33.49	1.46	0.69	1.98	0.49	1.35	−
111	TMV2	SB	13.84	4.60	43.66	32.69	1.65	0.61	2.14	0.48	1.34	−
112	OG52-1	SB	13.14	4.21	44.43	33.31	1.45	0.65	2.31	0.51	1.33	−
113	Tirupati1	SB	12.01	2.06	47.50	35.70	0.50	0.40	0.61	0.75	1.33	−
114	KRG1	SB	14.87	3.79	44.19	33.30	1.15	0.56	1.60	0.27	1.33	−
115	AK12-24	SB	13.79	4.58	43.73	33.20	1.64	0.65	1.97	0.41	1.32	−
116	LGN1	SB	12.89	3.68	44.57	33.96	1.34	0.71	2.36	0.50	1.31	−
117	Girnar1	SB	15.47	2.73	45.33	34.59	0.40	0.22	0.26	0.32	1.31	−
118	GJG31	SB	13.54	2.98	45.03	35.08	1.09	0.62	1.25	0.42	1.28	−
119	GG6	SB	13.95	4.28	43.16	33.82	1.39	0.65	2.23	0.53	1.28	−
120	SB XI	SB	13.76	4.44	44.02	34.53	1.16	0.46	1.30	0.13	1.27	−
121	Pratap Mungphali2	SB	13.45	3.43	44.48	35.10	1.15	0.51	1.40	0.48	1.27	−
122	Kadiri5	SB	13.32	3.44	43.19	34.32	1.48	0.77	2.58	0.91	1.26	−
123	GG8	SB	14.41	3.95	42.63	34.57	1.43	0.56	1.94	0.52	1.23	−
124	TMV9	SB	13.61	4.27	42.93	34.85	1.51	0.50	1.88	0.46	1.23	−
125	Kisan	SB	14.29	3.42	43.22	35.15	0.92	1.07	0.95	0.16	1.23	−
126	VRI3	SB	13.93	4.29	42.85	34.91	1.36	0.43	1.61	0.40	1.23	−
127	R8808	SB	13.73	2.16	43.56	35.72	1.17	1.01	1.95	0.41	1.22	−
128	Tirupati4	SB	13.38	5.20	41.50	34.18	1.87	0.66	2.63	0.56	1.21	−
129	Sp. Improved	SB	15.28	4.95	40.52	33.55	1.78	0.69	2.60	0.46	1.21	−
130	Tirupati2	SB	15.28	4.95	40.52	33.55	1.78	0.69	2.60	0.46	1.21	−
131	TMV (GN)13	SB	13.75	3.80	42.72	35.38	1.23	0.56	2.00	0.58	1.21	−
132	ALR3	SB	10.73	2.85	45.97	38.25	0.58	0.24	1.10	0.29	1.20	−
133	DH86	SB	13.35	3.82	42.78	35.64	1.36	0.73	1.82	0.51	1.20	−
134	ICGS44	SB	13.48	1.59	44.46	37.16	0.54	0.77	0.54	0.19	1.20	−
135	DH2000-1^*^	SB	13.17	4.66	42.12	35.31	1.49	0.58	1.91	0.77	1.19	−
136	Kadiri4	SB	13.98	4.07	41.54	35.08	1.39	0.77	2.27	0.91	1.18	−
137	CO1	SB	13.76	3.75	43.19	36.58	0.86	0.49	0.73	0.20	1.18	−
138	Co2	SB	13.82	2.95	44.04	37.32	0.65	0.36	0.37	0.13	1.18	−
139	DH101	SB	13.76	3.40	42.56	36.15	1.19	0.69	1.76	0.50	1.18	−
140	AK159	SB	13.14	3.63	42.38	36.01	1.40	0.74	2.15	0.53	1.18	−
141	JGN23	SB	13.34	4.01	41.76	35.56	1.49	0.63	2.70	0.52	1.17	−
142	BSR1	SB	13.70	2.40	41.51	35.36	1.29	1.14	3.16	1.45	1.17	−
143	JL220	SB	13.95	3.84	41.78	35.93	1.54	0.71	1.82	0.44	1.16	−
144	JCG88	SB	11.12	2.24	44.31	38.44	0.79	0.95	1.70	0.45	1.15	−
145	ICGS37	SB	14.66	2.41	42.06	36.52	1.19	0.95	1.48	0.54	1.15	−
146	JGN3	SB	13.43	3.78	41.42	36.28	1.47	0.75	2.42	0.46	1.14	−
147	GG3	SB	13.64	3.71	41.55	36.46	1.28	0.76	2.04	0.46	1.14	−
148	TMV7	SB	14.55	3.10	41.68	36.75	1.08	0.73	1.72	0.39	1.13	−
149	SG84	SB	14.59	1.93	42.81	37.96	0.25	0.33	0.30	0.11	1.13	−
150	ICGV00350	SB	13.37	1.66	42.61	37.87	0.92	1.01	1.99	0.57	1.13	−
151	ICGS11	SB	12.87	1.97	43.55	38.72	0.30	0.41	0.38	0.19	1.12	−
152	CO(GN)4	SB	14.20	3.08	41.44	36.94	1.17	0.84	1.92	0.41	1.12	−
153	Vemana/K134	SB	13.91	2.17	43.07	38.57	0.84	0.56	0.67	0.22	1.12	−
154	RG141	SB	14.87	2.68	41.42	37.50	0.65	0.40	0.63	1.00	1.10	−
155	Jawan	SB	14.29	3.30	42.18	38.67	0.36	0.28	0.30	0.17	1.09	−
156	GAUG1	SB	13.45	3.52	41.33	37.93	1.09	0.50	1.36	0.83	1.09	−
157	VRI2	SB	13.86	1.94	42.88	39.64	0.30	0.19	0.19	0.38	1.08	−
158	Dh8	SB	14.68	2.93	40.61	37.84	0.81	0.35	0.41	0.27	1.07	−
159	GG2	SB	14.81	2.02	42.08	39.51	0.36	0.29	0.28	0.30	1.07	−
160	JL286	SB	13.96	3.61	40.41	37.95	1.29	0.60	1.84	0.35	1.06	−
161	JL501	SB	12.44	2.81	41.73	39.36	1.04	0.62	1.64	0.36	1.06	−
162	TG3	SB	15.38	2.75	41.21	38.94	0.66	0.25	0.24	0.22	1.06	−
163	ICGV91114	SB	13.37	3.97	39.57	37.47	1.63	0.78	2.66	0.55	1.06	−
164	CO3	SB	14.42	2.93	38.69	36.90	1.41	1.04	3.25	1.33	1.05	−
165	ICGS1	SB	13.09	1.14	42.90	40.92	0.31	0.47	0.26	0.20	1.05	−
166	TG51	SB	14.21	3.53	40.36	38.74	1.07	0.48	1.25	0.37	1.04	−
167	Dh3-30	SB	13.70	2.47	39.94	38.35	1.26	1.03	2.46	0.79	1.04	+
168	TG37A	SB	13.75	2.81	40.62	39.05	0.93	0.62	1.61	0.62	1.04	−
169	ICGV86590	SB	11.60	2.30	41.52	40.14	1.09	0.89	1.91	0.44	1.03	−
170	TG22	SB	11.60	2.30	41.52	40.14	1.09	0.89	1.91	0.44	1.03	+
171	Pratap Mungphali1	SB	11.35	2.17	41.16	40.08	0.99	1.19	2.26	0.80	1.03	+
172	Kadiri6	SB	12.69	3.36	39.91	39.34	1.37	0.78	2.03	0.53	1.01	−
173	ICGV86031	SB	13.78	3.15	39.29	39.48	1.21	0.81	1.92	0.37	1.00	−
174	MH1	SB	13.58	3.02	37.87	40.65	1.23	0.80	2.24	0.61	0.93	−
	Mean^**^		12.26	2.93	49.43	31.29	1.08	0.79	1.60	0.48	–	−

Where; (+) Indicates ahFAD2A mutant allele; (−) Indicates ahFAD2A non-mutant allele; ND, Not Detected;

* Advance breeding lines;

** In the calculation of mean the data of “SunOleic95R” was not considered.

**Table 2:** **List of peanut wild relatives used (Bertioli et al., 2011; Gajjar et al., 2014)**.

**S. no**.	**Accession no**.	**Species**	**Section**	**Genome**	**Ploidy (*x* = 10)**
1	NRCG11802	*A. duranensis* Krapov and WC Gregory	*Arachis*	AA	2x
2	NRCG11837	*A. glabrata* Benth	*Rhizomatosae*	RR	2x
3	NRCG11786	*A. appressipila* Krapov and WC Gregory	*Procumbentes*	EE	2x
4	NRCG11793	*A. paraguariensis* Chodat and Hassl	*Erectoides*	EE	2x
5	NRCG12017	*Arachis correntina* (Burkart) Krapov and WC Gregory	*Arachis*	AA	2x
6	NRCG11781	*Arachis diogoi* Hoehne	*Arachis*	AA	2x
7	NRCG12989	*A. hermanii* Krapov and WC Gregory	*Erectoides*	EE	2x
8	NRCG12018	*A. batizocoi* Krapov and WC Gregory	*Arachis*	BB	2x
9	NRCG12046	*A. cardenasii* Krapov and WC Gregory	*Arachis*	AA	2x
10	NRCG12984	*A. cruziana* Krapov, WC Gregory and CE Simpson	*Arachis*	BB	2x
11	NRCG12031	*A. batizogaea* Krapov And A.Fernández	*Arachis*	BB	2x
12	NRCG12032	*A. rigonii* Krapov and WC Gregory	*Procumbentes*	EE	2x
13	NRCG11800	*A. monticola* Krapov and Rigoni	*Arachis*	AABB	4x
14	NRCG12029	*A. kretschmeri* Krapov. and WC Gregory	*Procumbentes*	EE	2x
15	NRCG17206	*A. marginata* Gardner	*Rhizomatosae*	EX	2x
16	NRCG17205	*A. prostrate* Benth	*Rhizomatosae*	EX	2x
17	NRCG12990	*A. pintoi* Krapov and WC Gregory	*Caulorhizae*	CC	2x
18	NRCG14871	*A. valida* Krapov and WC Gregory	*Arachis*	BB	2x
19	NRCG14855	*A. matiensis* Krappov	*Procumbentes*	PP	2x
20	NRCG14862	*A. stenosperma* Krapov and WC Gregory	*Arachis*	AA	2x
21	NRCG14868	*A. sylvestris* Chev	*Heteranthae*	Am	2x
22	NRCG468363	*Arachis* sp.	*Rhizomatosae*	–	2x

### DNA extraction

Two seeds of each genotype were grown in plastic pots filled with sand, under controlled conditions and leaf samples from the 10–15 days old seedlings were drawn for DNA isolation, using the cetyltrimethyl ammonium bromide (CTAB) extraction method (Cuc et al., 2008). However, for wild-species, leaf samples were taken from the peanut field gene-bank of DGR, Junagadh (India). The DNA quality was checked on agarose gel (0.8%, w/v) and quantification was done using NanoDropND-1000 (NanoDrop products, DE, USA) and working concentration was adjusted to 20 ng μL^−1^.

### Allele-specific PCR (AS-PCR) and cleaved amplified polymorphic sequences (CAPS) assays

For the identification of known mutations in the *ahFAD2* genes, all the genotypes were screened using known AS-PCR markers (Chen et al., 2010; Yu et al., 2013). Further, CAPS assays were also deployed to identify the zygosity of genotypes for *ahFAD2A* (448G>A as given by Chu et al., 2007) and *ahFAD2B* (441_442insA as given by Chu et al., 2009) mutations (Table S1).

For both, AS-PCR and CAPS analysis, the PCR mixtures (10 μl) contains, template DNA (1 μl, 20 ng), 5X PCR buffer (2 μl, Promega, USA), 25 mM MgCl_2_ (0.8 μl, Promega, USA), 2 mM dNTP (0.7 μl, Thermo Fisher Scientific, USA), primers (0.5 μl, 25 p moles), 5U Taq polymerase (0.2 μl, Promega, USA) and sterile double distilled water (4.3 μl). Amplification was performed in a thermal cycler (Eppendorf, USA) using thin walled 96 wells PCR plates (Sorenson™ Bioscience, USA).

The touchdown PCR was done with initial denaturation at 94.0°C for 3 min and then 5 cycles of the following: 94.0°C for 30 s (−1.0°C reduction per cycle), 65–60°C for 30 s and 72.0°C for 1 min. This was followed by another 35 cycles of 94.0°C for 30 s, 60.0°C for 30 s and 72.0°C for 1 min of denaturation, annealing and extension, respectively. Final extension was 72.0°C for 10 min. Amplification was performed twice and amplified products were analyzed using 2% agarose gel in 1 × TBE buffer at 225 volts for about 2.5–3.0 h and stained with ethidium bromide. The gels were documented in automated gel documentation system (Fujifilm FLA-5000) and scored.

For CAPS analysis of *ahFAD2A* and *ahFAD2B* mutations, the amplified products (10 μl) of 826 and 1200 bp were digested using 0.4 μl each, of *Hpy*99I and *Hpy*188I (0.4 U; New England Biolabs, MA) restriction enzymes, respectively, in 2.0 μl digestion buffer, 2.0 μl BSA (0.1%, Takara, Japan) and 5.6 μl distilled water. The restriction digestion of *ahFAD2A* and *ahFAD2B* gene specific amplicon was done at 37°C for 4 h, and 37°C for overnight with *Hpy*99I and *Hpy*188I enzymes, respectively.

### Fatty acid profiling

The fatty acids were analyzed using gas chromatography system (Thermo fisher, Trace GC 1100) equipped with flame ionization detector (FID). The fatty acid methyl esters were passed through capillary column (TR-wax), and the esters of fatty acids were estimated (Misra and Mathur, 1998). The inlet, FID detector were set to 240°C and oven at 190°C whereas carrier gas (nitrogen) and fuel gas (hydrogen) were maintained at 30 mL per min. Total run time for each sample was 12 min and the peaks were identified by comparison to a FAME standard mix RM-3 (Sigma-Aldrich, St. Louis, MO).

Pearson's coefficient analysis was performed using SPSS 15.0 software to determine correlations among different fatty acids. An analysis of variance was performed on mean values to test the significant differences in major fatty acids among the mutant and non-mutant genotypes using Tukey's multiple comparison test (Brown, 1979).

### Single nucleotide polymorphism (SNP) analysis of *ahFAD2A* gene

A set of 30 peanut genotypes were selected based on *ahFAD2*A gene mutation and O/L ratio so as to find the allelic variations as SNPs (Table S2). The functional domain flanking sequence information of desaturase enzyme was used to design *FAD2* gene specific primers, using Primer3plus software (http://primer3plus.com), with expected amplicon size of 1148 bp (Table S1). PCR reaction conditions were same as of AS-PCR and CAPS assays; except, it is carried out in a final volume of 20 μl. The PCR products from each sample were visualized by agarose gel electrophoresis.

#### Cloning of full length *ahFAD2* gene

The *ahFAD2* gene specific bands were excised and purified using QIAquick gel extraction kits (QIAGEN), and used for direct sequencing as-well-as cloning, using pGEM®-T Easy cloning kit (Promega). The amplified products were ligated to pGEM®-T easy vector, using T_4_ DNA ligase (4°C), and kept overnight. Transformations were carried out via conventional heat shock method into competent *E. coli* (Dh5-Alpha) cells, and selection of transformed cells was done on LB/ampicillin/IPTG/X-Gal plates. Positive colonies were identified using vector specific (SP6 and T7 primers) and gene specific primers. Positive colonies were grown overnight and plasmid DNA was isolated using QIAgen kit and utilized for DNA sequencing. The sequencing was performed using SciGenome's (Kerala, India) Sanger sequencing services using ABI 3730Xl platform.

#### Sequence analysis

The raw sequences were edited and aligned using CAP3 programme (Huang and Madan, 1999), to generate consensus sequences. Further, the chromatograms were also checked manually, so as to find any miscalled bases in the identified SNPs. The *ahFAD2A* and *ahFAD2B* gene sequences were distinguished based on the previously reported SNPs. Subsequently, both *ahFAD2A* and *ahFAD2B* sequences were aligned using ClustalW programme. The phylogenetic tree was constructed using Neighbor-Joining method of MEGA 6 program (Tamura et al., 2013), and visualized using online tool iTOL (Letunic and Bork, 2011). The haplotype numbers, haplotype diversity, nucleotide diversity, non-synonymous (K_a_) to synonymous (K_s_) ratio were generated using DnaSP 5.10 software (Librado and Rozas, 2009).

## Results

### Detection of mutations in *ahFAD2* genes using AS-PCR and CAPS assays

**Figure 1:**
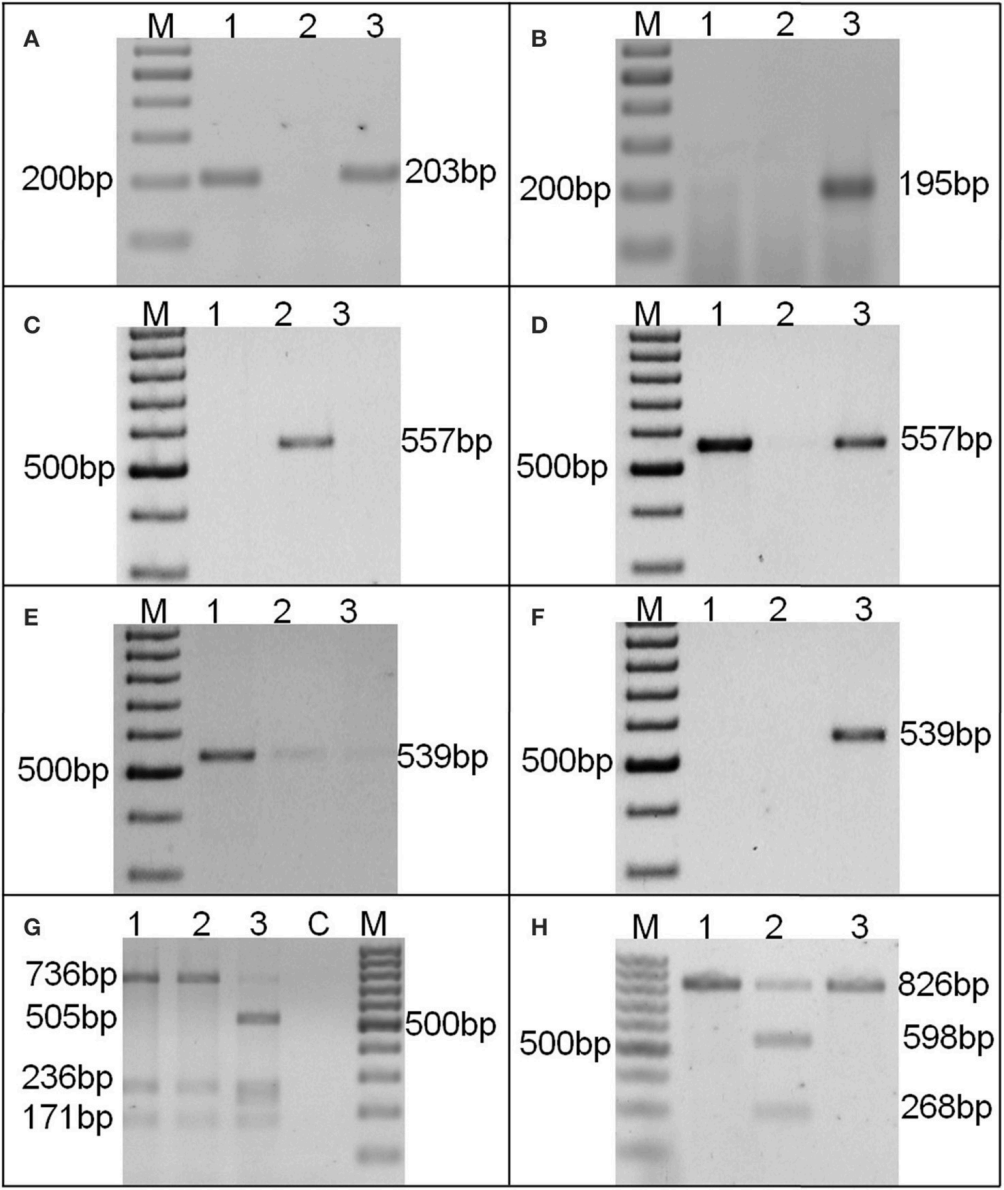
**A representative picture of AS-PCR (A–F) and CAPS assays (G,H) AS-PCR assay (Chen et al., 2010) showing (A) ***ahFAD2A*** mutant allele specific 203 bp amplification; (B) ***ahFAD2B*** mutant allele specific 195 bp amplification; AS-PCR assay (Yu et al., 2013) showing (C) ***ahFAD2A*** non-mutant allele specific 557 bp amplification; (D) ***ahFAD2A*** mutant allele specific 557 bp amplification; (E) ***ahFAD2B*** non-mutant allele specific 539 bp amplification and (F) ***ahFAD2B*** mutant allele specific 539 bp amplification; Amplicons generated by CAPS assay for (G) ***ahFAD2***B and (H) ***ahFAD2A*** alleles**. Lanes: 1: GG20, 2: Kadiri3, 3: SunOleic95R, M: 100 bp DNA ladder.

The AS-PCR assay (Chen et al., 2010) for substitution allele-specific primer combination, amplified 203 bp amplicon in 80 genotypes, confirming a single bp substitution (G:C/A:T) at 448 position in *ahFAD2A* allele (Figure 1A). While, insertion (A:T at 442 bp position) allele-specific primer combination for *ahFAD2B* allele, did not amplify in any genotypes. Only a known mutant genotype, SunOleic95R showed a 195 bp amplicon (Figure 1B). In addition, the primer combination F435-F and F435IC-R was used as internal control and primer combination F435-F and F435WT-R amplified a 193 bp amplicon in all the genotypes.

Since, the AS-PCR assay of Chen et al. (2010) was unable to identify the zygosity of both *ahFAD2A* and *ahFAD2B* genes; therefore, AS-PCR assay of Yu et al. (2013) was used (Table S1). All the 80 genotypes were found having *ahFAD2A* mutant allele in homozygous condition (*ol1ol1/Ol2Ol2*) (Figures 1C,D). The result of *ahFAD2B* gene revealed non-mutant gene specific amplification in all the genotypes (Figures 1E,F).

Further, CAPS assay was also used for the identification of 448G>A mutation in *ahFAD2A* gene and 441_442insA mutation in *ahFAD2B* gene (Table S1). Full-length amplification of *ahFAD2A* gene generated an 826 bp product, which was digested into 598 and 228 bp fragments (with *Hpy*991 enzyme) in 93 genotypes, predicting their wild-type nature. However, PCR products of 80 genotypes remain undigested, showed mutation in the *ahFAD2A* gene. Moreover, a 1200 bp band of *ahFAD2B* gene, digested into 736, 263, 171 bp bands with enzyme *Hpy*1881, indicating its non-mutant nature. Only SunOleic95R produced banding pattern akin to *ahFAD2B* mutant allele (Figures 1G,H). All the 22 wild-species (Table 2), when tested using AS-PCR assay (Chen et al., 2010), showed the presence of non-mutant alleles.

### Pedigree analysis of indian peanut cultivars for *ahFAD2A* gene

Since, 46% of the Indian peanut cultivars recorded *ahFAD2A* mutation; therefore, the pedigree was also analyzed so as to find the association of *ahFAD2A* mutant allele with its parents. Out of 26 varieties having *ahFAD2A* mutant allele, both the parents of 3 varieties and one parent of 23 varieties found containing mutant *ahFAD2A* gene. Moreover, in 34 non-mutant varieties, 12 had either non-mutant *ahFAD2* gene in both the parents or were derived from the selection. The details of all the genotypes are given in Table S3.

Cultivated peanut is known to have an extremely narrow genetic base due to the inbreeding among a few select parental lines in commercial breeding programs (Nigam, 2000; Simpson et al., 2001). This was found true, when pedigree of 167 Indian cultivars were critically analyzed. It is found that 08 genotypes were most frequently (4 times or more) used as one of the parent, leading to the release of 59 cultivars (Table 3). Thus, there is need of better utilization of existing variability in Indian peanut improvement programs.

**Table 3:** **List of varieties frequently used in the Indian varietal development programme**.

**S. no**.	**Genotypes/varieties**	**No of times used for variety development**	**Varieties developed**
1	JL24	14	VRI2, GG3, K134, Tirupati4, GG5, JGN39, CO3, AK159, Kadiri5, Kadiri6, AK303, LGN1, JGN23 and GG8
2	M13	6	Somnath, B95, M335, GG11, BAU13 and Girnar2
3	ICGS11	5	R8808, AK265, Girnar3, R2001-2 and R2001-3
4	GAUG10	4	GG20, GG11, GG13 and GG12
5	J11	4	GG2, Jawan, VRI3 and JCG88
6	Spanish Improved	4	TG1, DH330, Kisan and TG3
7	Robut 33-1	18	ICGS11, Kadiri3, ICGS44, ICGS1, GG20, ICGS5, TG22, VRI3, RG141, ICGS37, DRG17, DRG12, BSR1, LGN2, ALR3, GG14, HNG10 and Girnar2
8	Chico	4	ICGS76, GG4, R8808 and TG51
	Total	59	–

### Fatty acid profiling

In the 174 peanut genotypes studied, oleic (C18:1), linoleic (C18:2) and palmitic (C16:0) acids were the major fatty acids, of which, the first two constitute about 80% of total fatty acids. Behenic (C22:0), stearic (C18:0), arachidic (C20:0), gadoleic acid (C20:1), lignoceric (C24:0), were also present in smaller amounts in all the genotypes (Table 1 and Figure S1). Myristic acid was found in 19 genotypes; interestingly all of which belongs to Spanish bunch group (Table S4).

Fatty acid profiling revealed highest and lowest contents of oleic acid in TMV10 (66.57%) and MH1 (37.87%); while, linoleic acid in ICGS1 (40.92%) and GJG22 (17.41%), respectively (Table 1). Similar trends were obtained for ICRISAT mini-core collections; where highest, 71% of oleic acid and lowest 15.81% linoleic acid were reported (Mukri et al., 2012). The average oleic and linoleic acid contents were recorded as 49.61% and 31.15%, respectively; while, Bishi et al. (2015) reported an average of 46.47% oleic acid and 34.51% linoleic acid in 41 Indian peanut cultivars.

The mean of oleic acid content was found significantly higher for 80 *ahFAD2A* mutant lines (55.91%), than the 93 non-mutant lines (43.85%). Fourteen mutant genotypes for *ahFAD2A* gene recorded more than 60% oleic acid content (Table 1); thus, for increasing the O/L ratio, these genotypes should be the first choice. The high oleate trait can be combined with other traits like disease resistance, for which foliar disease resistance genotypes *viz*. GPBD4 and AK265 as well as peanut bud necrosis resistant genotype R2001-3, could be taken into breeding program (Table 1). Since, these genotypes already have one mutant allele (*ahFAD2A*) in homozygous condition; therefore, another mutant gene (*ahFAD2B*) can be easily transferred using marker assisted backcross breeding (MABB) in allotetraploid peanut.

Among the genotypes studied, SunOleic95R, TMV10, HNG10 and GG16 were found to have less than 9% pamitic acid, which along with high oleic and low linoleic acid can make the peanut oil healthier for consumers. The genotypes, GG13, BAU13, ICGS76 had low oil (<45%), high sucrose (>6%), high oleic acid (>58%), and high O/L ratio (>2%) which is highly desired combination for confectionery peanut industry.

### Classification of mutant varieties on the basis of botanical types

The 173 cultivated peanut genotypes studied, belong to two subspecies *viz*. ssp. *fastigiata* (102) and ssp. *hypogaea* (71); which recorded 17 and 62 mutant, while 85 and 8 non-mutant genotypes, respectively. In general, mutant lines showed higher mean O/L ratio than the non-mutant botanical types. Interestingly, no genotype of Valencia group recorded mutation in *ahFAD2A* gene, while all the genotypes of Virginia runner group showed substitution mutation (Table 4). Mutant Virginia runner genotypes, recorded mean O/L ratio of 2.34 with highest oleic (57.23%), lowest palmatic (10.75%) and linoleic acid contents (25%). Highest range of O/L ratio was observed in Virginia bunch mutant type (1.38–3.76); whereas, ssp. *hypogaea* recorded high oleic, low linoleic and palmitic acid than the ssp. *fastigiata* (Table 4). These results are in accordance with Wang et al. (2013b), Mukri et al. (2012), and Wang et al. (2009).

**Table 4:** **Classification of peanut genotypes on the basis of botanical types and major fatty acid composition**.

**Botanical type**	**Habit group**	***ahFAD2A* status**	**No. of genotypes**	**Palmitic acid (%)**	**Oleic acid (%)**	**Linoleic acid (%)**	**Mean O/L ratio**	**Range of O/L ratio**
ssp. *fastigiata*	Spanish Bunch (vars. *vulgaris*)	Mutant	17	11.37	51.96	29.86	1.84	1.00–2.90
		Non-mutant	80	13.45	43.90	35.29	1.26	0.93–2.30
	Valencia (vars. *fastigiata*)	Mutant	Nil	–	–	–	–	–
		Non-mutant	5	12.57	41.53	38.05	1.10	0.93–1.31
ssp. *hypogaea*	Virginia Runner (vars. *hypogaea*)	Mutant	31	10.75	57.23	25.0	2.34	1.55–3.33
		Non-mutant	Nil	–	–	–	–	–
	Virginia Bunch (vars. *hypogaea*)	Mutant	32	10.98	56.77	27.08	2.25	1.38–3.76
		Non-mutant	08	12.92	44.68	35.21	1.29	1.04–1.71
	Total/mean		173	12.26	49.43	31.29	1.69	0.93–3.76

### Correlations studies and effect of *ahFAD2A* mutation on fatty acid composition

In correlation studies, oleic acid was found negatively correlated with all the SFAs including palmitic acid (*r* = −0.87), which most likely represents an increased rate of palmitic acid elongation to stearic acid, with rapid desaturation to oleic acid via Delta-9 desaturase (Wang et al., 2015a). A significant positive correlations are observed between linoleic acid and palmitic acid (*r* = 0.78), stearic acid and arachidic acid (*r* = 0.68) and also between arachidic acid and behenic acid (*r* = 0.78) (Table 5).

**Table 5:** **Correlation coefficients among fatty acid components of different peanut genotypes**.

	**Palmitic acid (C16:0)**	**Stearic acid (C18:0)**	**Oleic acid (C18:1)**	**Linoleic acid (C18:2)**	**Arachidic acid (C20:0)**	**Gadoleic acid (C20:1)**	**Behenic acid (C22:0)**	**Lignoceric acid (C24:0)**
Palmitic acid	1							
Stearic acid	0.38^**^	1						
Oleic acid	−0.87^**^	−0.33^**^	1					
Linoleic acid	0.78^**^	0.13	−0.96^**^	1				
Arachidic acid	0.15^*^	0.68^**^	−0.22^**^	0.02	1			
Gadoleic acid	−0.50^**^	−0.26^**^	0.40^**^	−0.44^**^	0.28^**^	1		
Behenic acid	0.05	0.44^**^	−0.22^**^	0.05	0.78^**^	0.44^**^	1	
Lignoceric acid	0.55	−0.09	−0.15^*^	0.07	0.25^**^	0.16^*^	0.33^**^	1

Where,

* and ** denotes significance level at 0.05%, 0.01%, respectively.

**Figure 2:**
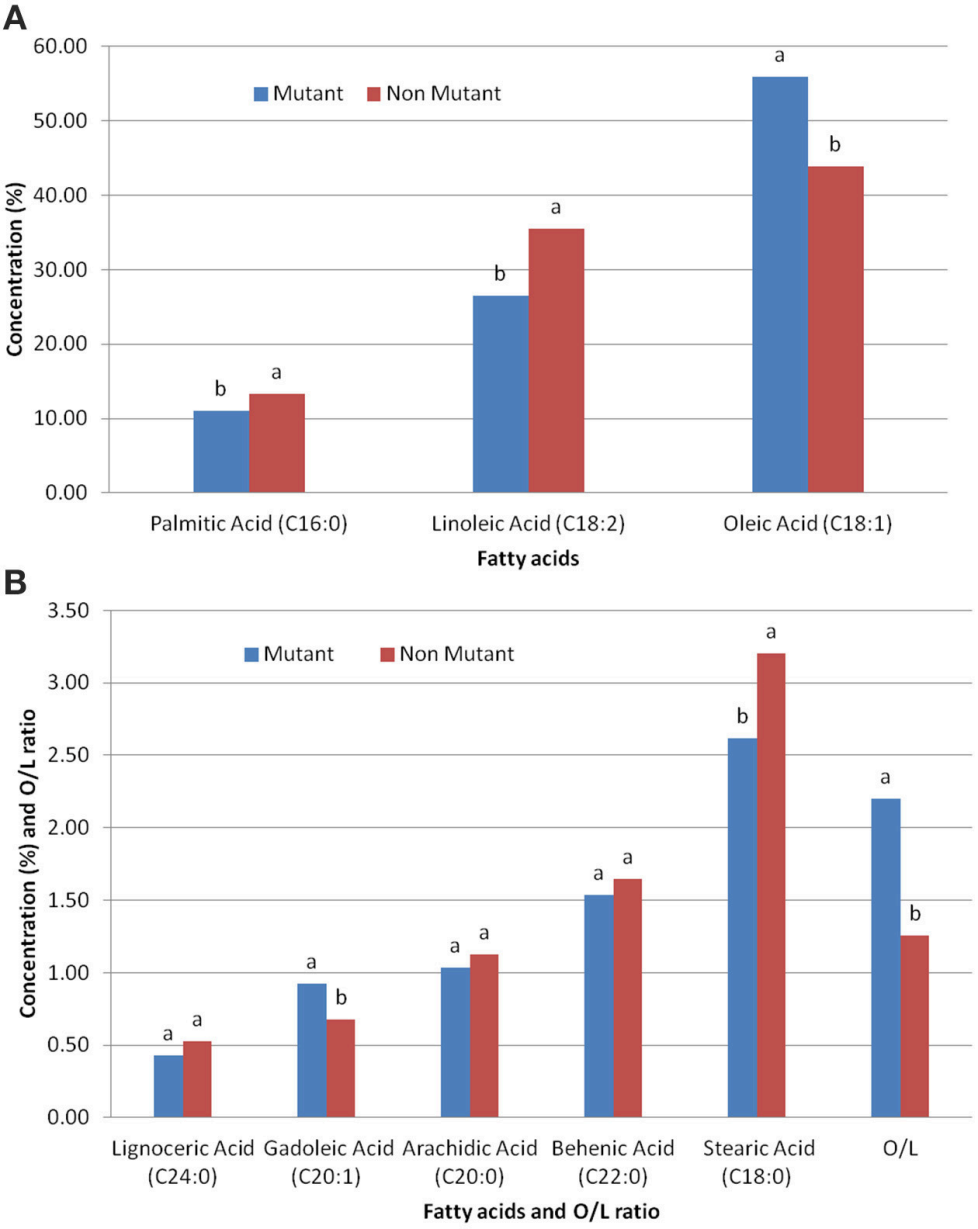
**Effect of ***ahFAD2A*** mutant allele on (A) various fatty acids; (B) various fatty acids and O/L ratio**.

The effect of *ahFAD2A* gene mutation, on both O/L ratio, and other fatty acids composition, revealed significant reduction in the palmitic acid, stearic acid and linolenic acid contents in mutant genotypes. However, significant increase in the oleic acid, gadoleic acid and O/L ratio was observed. No significant difference was recorded for the fatty acids like arachidic acid and behenic acid in both, mutant and non-mutant genotypes (Figure 2).

### SNP analysis of *ahFAD2* gene in selected peanut genotypes

Since a gradient in the O/L ratio was observed in the genotypes studied; therefore, a set of 30 genotypes, having 17 mutant and 13 non-mutant lines for *ahFAD2A* gene were selected for further sequencing of the *ahFAD* gene (Table S2). To amplify the full functional domain of *ahFAD2A* and *ahFAD2B* genes, the primers were designed using GenBank accession >gi|307697073|gb|HM359250.1| (Table S1), which amplified an 1148bp amplicon. Both, direct sequencing of PCR products, and clone based sequencing was performed for both *ahFAD2A* and *ahFAD2B* genes.

The chromatogram analysis of PCR products revealed double peaks at 19 positions, since it consisted of amplicons from both *ahFAD2A* and *ahFAD2B* genes (Figure S2). Previously, overlapped peak were reported to identify the heterozygous state of allele (Wang et al., 2011b; Yu et al., 2013). To distinguish the sequences of A and B genomes in the genotypes studied, 1148 bp gene specific amplicons were cloned and sequenced. Further, the sequence information pertaining to *ahFAD2A* (GenBank accession no. HM359250.1 and GQ412349) and *ahFAD2B* (GenBank accession No. HM359251 and HM359252.1) genes were used to identify the sequences of *ahFAD2A* and *ahFAD2B* genes in the genotypes studied (Tables S5, S6).

**Figure 3:**
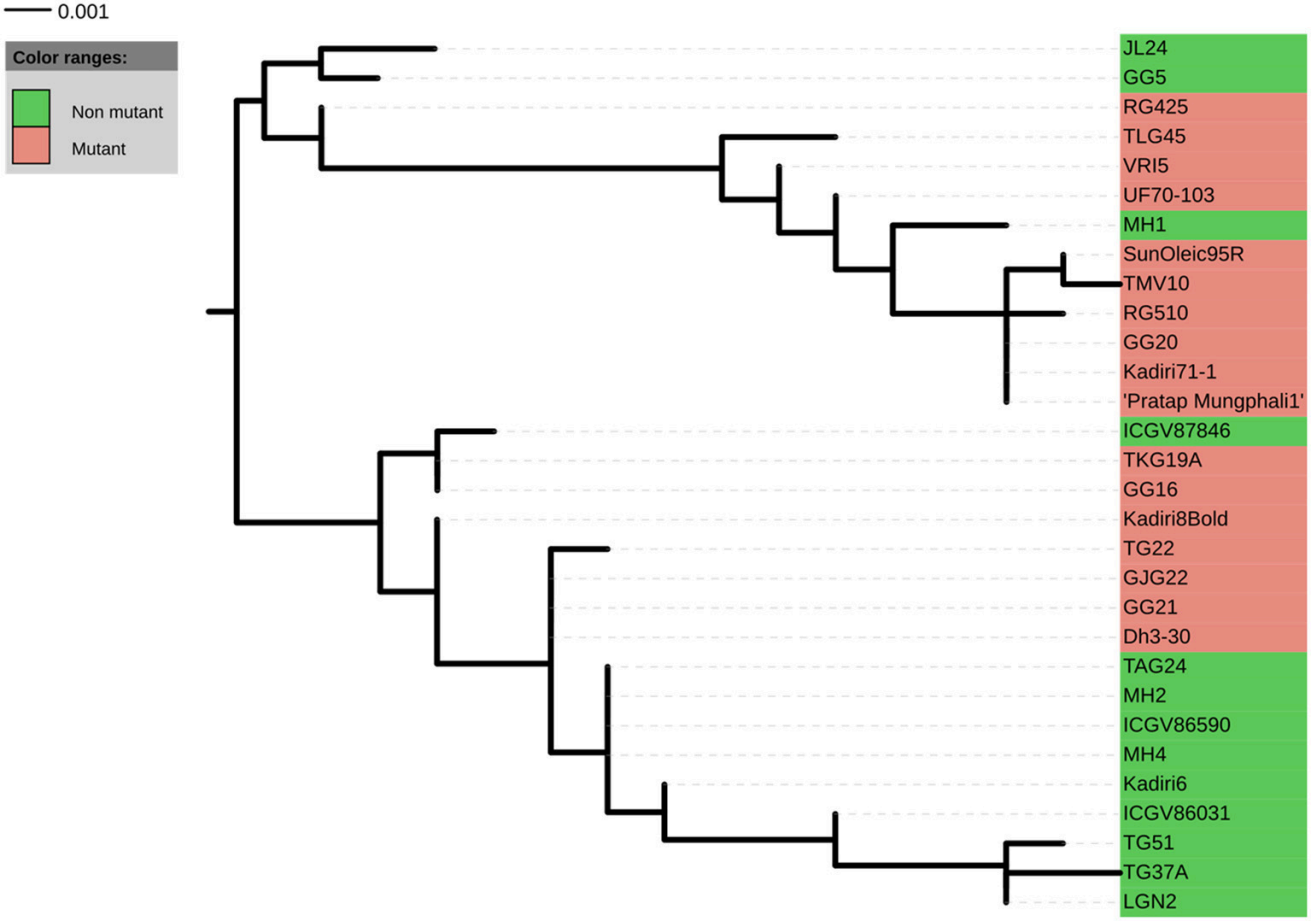
**Cladogram generated from cluster analysis using ***ahFAD2A*** nucleotide sequence data of 30 genotypes**.

**Figure 4:**
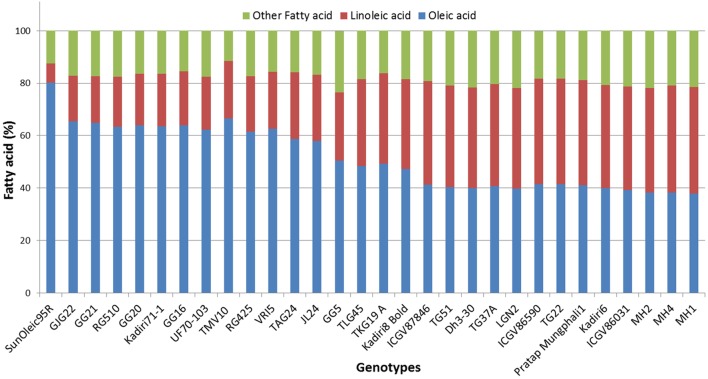
**Fatty acid profile of selected peanut genotypes representing high, medium and low oleic acid to linoleic acid ratio**.

A total of 22 and 21 haplotypes numbers (H) with haplotype diversity (H_d_) of 0.970 and 0.952 were detected in *ahFAD2A* and *ahFAD2B* genes, respectively. The nucleotide diversity (P_i_) for *ahFAD2A* and *ahFAD2B* were 1.05% and 0.95%, respectively. In present study, the ratio of non-synonymous substitution (K_a_) to synonymous substitution rates (K_s_) or K_a_/K_s_ ratio of 0.36 and 0.39 were obtained for *ahFAD2A* and *ahFAD2B* gene sequences, respectively (Table 6). Combined phylogenetic analysis of SNPs present in the *ahFAD2A* and *ahFAD2B* genes, among 30 genotypes revealed two major clusters. Cluster 1 comprised of mainly mutant genotypes except MH1 which is a non-mutant genotype. However, cluster 2 consisted of both mutant and non-mutant genotypes. Overall, no clear clustering pattern has been observed based on the SNP sequences of desaturase gene (Figure 3). Similar pattern was obtained when clustering was done using *ahFAD2A* and *ahFAD2B* genes separately (Figures S4, S5). Fatty acid profiles of selected genotypes representing high, medium and low, oleic, linoleic and other fatty-acids are presented in Figure 4.

**Table 6:** **Summary of nucleotide polymorphism analysis of ***ahFAD2A*** gene among selected peanut genotypes**.

**Gene**	**Genome**	**Haplotypes**		**P_i_**	**Synonymous SNPs (K_s_)**		**Non-synonymous SNPs (K_a_)**				**Total number of SNPs**	**K_a_/K_s_ ratio**	**InDel**
		**H**	**H_d_**		**A/G**	**T/C**	**A/T**	**G/C**	**A/C**	**G/T**			
*ahFAD2*	A	22	0.970 ± 0.018	0.0105	4	10	1	1	2	1	19	0.36	0
	B	21	0.952 ± 0.027	0.0095	3	10	1	1	2	1	19	0.39	1

Where, H, Haplotype numbers; H_d_, Haplotype diversity; P_i_, nucleotide diversity.

## Discussion

Out of 167 varieties and 6 advanced breeding lines studied; 77 (46%) and 3 (50%) genotypes, respectively, showed the presence of *ahFAD2A* muation, while none had *ahFAD2B* mutant allele. Similarly, in US mini-core collections, 44% (Wang et al., 2011b), 31.6% (Chu et al., 2007) and 41% (Wang et al., 2013b); while in ICRISAT mini-core collections, 49.5% (Mukri et al., 2012) *ahFAD2A* mutant allele was reported. But, none found having *ahFAD2B* mutant allele. However, all the 22 wild-species, showed absence of mutation in the both *AhFAD2A* and *ahFAD2B* alleles. Similar results were also observed in 27 *Arachis duranensis* accessions (Chu et al., 2007), and in 39 wild-species including the two putative progenitors of the cultivated peanut (Wang et al., 2010). Therefore, it seems that the substitution mutation in *ahFAD2* genes might have occurred after the polyploidization of cultivated peanut (Chu et al., 2007; Wang et al., 2010); and *ahFAD2A* mutant allele appears to have arisen before the global distribution of peanut from its center of origin (South America). This could be the reason that, only *ahFAD2A* mutant allele has been reported in a range of genotypes across the world (Chu et al., 2007; Wang et al., 2011b, 2013b; Mukri et al., 2012); and the absence of *ahFAD2B* mutation, might be due to its recent origin (Norden et al., 1987).

Since, the AS-PCR assay of Chen et al. (2010) is unable to detect the zygosity of the genes; and even in CAPS assay for *ahFAD2A* mutation, problem of incomplete digestion of PCR product was observed (Jung et al., 2000b; Chu et al., 2007). Therefore, the zygosity of *ahFAD2A* gene was studied using AS-PCR assay of Yu et al. (2013); which identified all the 80 mutant genotypes as homozygous. The results are expected, since all these lines were highly stable in nature. Moreover, for *ahFAD2B* gene, both AS-PCR (Yu et al., 2013) and CAPS (Chu et al., 2009) assays were found efficient for the zygosity detection.

Pedigree analysis has clearly revealed that, the mutant *ahFAD2A* gene has moved from its parents to the varieties. However, in the variety Kadari9, although both the parents were found non-mutant; but, it still had *ahFAD2A* mutation. The possible explanations could be the origin of a spontaneous mutation, although it seems a rare possibility in a varietal cross. On the contrary, a non-mutant variety, MH1 was developed through selection of Faizpur1-5, having *ahFAD2A* mutant gene (Table S3). Thus, considering the polyploid nature of peanut, it might take several generations of inbreeding to have a plant without a mutant allele. Another possibility could be that, during the development of these varieties, the lines might have accidently crossed with some other mutant and non-mutant lines, respectively. This could have resulted in fixation of mutant and non-mutant allele in Kadiri9 and MH1, respectively, during the course of selection. This needs further confirmation, using in-depth molecular diversity studies. Overall, it is clearly revealed that, large number of Indian peanut cultivars share common parent, which was also shown by the presence of *ahFAD2A* mutant allele in 46% of the varieties.

In this study, the analysis of peanut botanical types, reported more frequent *ahFAD2A* mutation in ssp. *hypogaea* (89%) compared to ssp. *fastigiata* (17%). Similarly, Mukri et al. (2012) reported 84.52% of the ICRISAT mini-core accessions belonging to ssp. *hypogaea* and 19.39% of ssp. *fastigiata* types carried the *ahFAD2A* mutant allele. Wang et al. (2013b) also reported 33.82, 6.37, 1.96, and 0% *ahFAD2A* mutation in vars. *hypogaea, vulgaris, fastigiata* and *peruviana*, respectively. This is also in agreement with Chu et al. (2007) and Wang et al. (2009), where in the U.S. peanut mini-core collections more frequent *ahFAD2A* mutation was reported in ssp. *hypogaea* than in ssp. *fastigiata*. The pedigree analysis of mutant genotypes of ssp. *fastigiata* revealed that, only five genotypes were derived from TG1, and one from TMV10, as a parent having *ahFAD2A* mutation (Table S3). Therefore, the occurrence of very low frequency of *ahFAD2A* mutation in ssp. *fastigiata* can be explained by the hypothesis of origin of ssp. *hypogaea* from ssp. *fastigiata* (Singh, 1988).

The mutant genotypes showed significantly higher average O/L ratio (2.20) than the non-mutants genotypes (1.26), and average O/L ratio of all the genotypes was 1.69 with a range of 0.93 (MH1) to 3.76 (GJG22). Similarly, in *ahFAD2A* mutant germplasm lines, the range of mean O/L ratio recorded was 1.11–6.93 with an average of 2.43, whereas, in the non-mutant accessions it was 1.26 with a range of 0.77–2.55 (Mukri et al., 2012). Majority of the *ahFAD2A* mutant genotypes were found having O/L ratio above 1.5, while non-mutant lines had less than that, with some exceptions (Table 1). Nearly similar results were reported by Wang et al. (2013b). This means, genotyping for *ahFAD2A* mutant allele using molecular makers is quite reliable and robust method to discriminate or identify the genotypes with high oleic acid. Overall, three genotypes *viz*. GG20, ICGV86325 and BG2 were found having high oil (>48%), low behenic acid (<1%), high oleic acid (>58%) (Table 1), which are more desirable traits for the oil industry (Grosso et al., 2000).

Highly negative correlation was observed between oleic acid and linoleic acid (*r* = −0.96), as also observed by Wang et al. (2013b, 2015a). The accumulation of oleic acid might have led to the increase in gadoleic acid content, which is formed from it. On the other hand, since more palmitic acid (C16:0) is mobilized toward the oleic acid (C18:1), it may have led to the decrease in the contents of palmitic acid, stearic acid, arachidic acid, behenic acid and lignoceric acid. This could be the reason for the negative correlation of oleic acid with all these SFAs (Wang et al., 2015a).

As reported in this study, other results also showed two mutant fatty acid desaturase (*ahFAD*) alleles, controlling three major fatty acids *viz*. oleic, linoleic and palmitic acid (Wang et al., 2013b, 2015a; Pandey et al., 2014; Janila et al., 2016). Some reduction in the production of linoleic, palmitic, stearic and arachidic acid, while no influence of these mutant alleles on the behenic and lignoceric acid was observed as also reported by Wang et al. (2013b, 2015a). Since, gadoleic acid is formed from oleic acid; therefore, an increase in the oleic acid content results in an increase in gadoleic acid content. On the other hand, since more palmitic acid is mobilized toward the oleic acid formation, the oleic acid acts as a signal to trigger a negative feedback loop to deal with an excess of SFAs, which might have resulted in decrease in the contents of palmitic acid, stearic acid, arachidic acid, behenic and lignoceric acid as also reported by Lim et al. (2013) and Harvey et al. (2010).

Interestingly, the *ahFAD2B* gene of all the peanut genotypes, found transcribing full 379 amino acid protein functional domains; whereas, the *ahFAD2A* gene of 19 genotypes showed stop codons (Figure S3). Similarly, five partial truncated sequences were also identified by Wang et al. (2015e), which might have translated a truncated fatty-acid desaturase protein. The changes in coding sequences might have caused varying efficiency of metabolic processes of fatty acid desaturase enzyme formation. Recently, Wang et al. (2015e) identified six distinct novel members in the *ahFAD2* gene family involved in the regulation of linoleic acid synthesis. Among these, *ahFAD2-2, ahFAD2-3* and *ahFAD2-4* variants, displayed a high degree of sequence similarity with the known peanut *ahFAD2* sequences. As we have used single primer pair, targeting coding regions of *ahFAD2* gene, the resulting sequences may be representing different members of *ahFAD2* gene. This might be one of the possible reasons for the mixing of both mutant and non-mutant genotypes in the cladogram, which needs further confirmations.

Mutations in general and SNPs in particular are the major driving forces causing genetic variation in peanut *ahFAD* genes. An understanding of the mutations in these genes and proteins in peanut is of prime importance due to its fundamental role in regulating the O/L ratio. The values of K_a_/K_s_ give a clear picture into the evolution of *ahFAD* gene (Hurst, 2002) and the observed K_a_/K_s_ ratio of <1 for both *ahFAD2A* and *ahFAD2B* gene sequences of the Indian peanut genotypes, implies that most amino acid substitutions have been eliminated by the strong purifying or stabilizing selection. It is also reported that the proteins with rigorous functional or structural requirements are subject to such negative selection pressure, resulting in smaller numbers of amino acid changes and tend to evolve gradually (Kondrashov et al., 2002). Thus, our analysis showed that the K_a_/K_s_ ratio can certainly be a practical and robust measure to infer the direction and magnitude of natural selection acting on any gene in peanut.

The O/L ratio among the *ahFAD2A* mutant genotypes ranged from 1.03 to 3.76 (Table 1). Similarly, Mukri et al. (2012) and Wang et al. (2010) have recorded O/L ratio in the range of 1.11–6.93 and 1.86–3.42, respectively. Since, the genetic backgrounds for each genotype in our study are different; this might have affected the levels of oleic acid and linoleic acid. Moreover, reports on recombinant inbred lines (RILs) also revealed considerable phenotypic variations for different fatty acids (Sarvamangala et al., 2011; Pandey et al., 2014; Wang et al., 2015a). Even for the introgression lines (ILs) with homozygous *ahFAD2* mutant alleles, considerable variations in the oleic and linoleic acid content from 62 to 82% and 02 to 20%, respectively, was recorded (Janila et al., 2016).

The double mutant line SunOleic95R is reported to have 79–81% oleic acid and 2.5 to 4.7% linoleic acid under US conditions (Gorbet and Knauft, 1997; Barkley et al., 2010, 2011). However, the seeds of same line which were grown under Junagadh, India (21.31° N, 70.36° E, 200 m above mean sea level, AMSL) conditions, recorded 80.18% oleic acid and 7.34% linoleic acid. Similarly, Janila et al. (2016) also reported 78.3% oleic acid and 6% linoleic acid for this genotype at ICRISAT-Patancheru, India (17.50° N, 78.27° E, 545 m AMSL). The oleic acid content seems nearly the same under both Indian and US environmental conditions, while linoleic acid content was relatively higher under Indian conditions. This clearly implies the involvement of other factors, influencing the production of linoleic acid.

The O/L ratio in two non-mutant genotypes, JL24 and TAG24 in the present investigation was recorded as >2.0; however, Bishi et al. (2015) and Mukri et al. (2012) recorded, <1.5 for these genotypes. On the other hand, Mondal et al. (2010) for the genotype TAG24 recorded O/L ratio as 1.85. On the similar note, normal oleic seeds were also identified in seed-lots of the high-oleic peanut cultivar “Brantley” (Chamberlin et al., 2011). The peanut fatty-acid compositions were found influenced by the genetic factors and its interaction with environment (Andersen and Gorbet, 2002; Singkham et al., 2010) and also by various environmental factors like temperature (Sun et al., 2014), growing season (Singkham et al., 2010), and maturity (Hinds, 1995).

Wang et al. (2013a) reported that 60% of the variation in oleic or linoleic acid content can be explained by the genotypic effect of *ahFAD2A* and *ahFAD2B* genes. Pandey et al. (2014) found two marker intervals with phenotypic variance (PVE) i.e., *ahFAD2B* gene (26.54, 25.59, and 41.02% PVE) and *ahFAD2A* gene (8.08, 6.86, and 3.78% PVE) for oleic acid (C18:1), linoleic acid (C18:2), and oleic/linoleic acid ratio (O/L ratio), respectively. All these reports indicate the presence of other factors, regulating the O/L flux. Recently, some novel members of a*hFAD2* genes with varying expressing levels were identified in peanut. Besides, presence of other candidate genes controlling oleate levels in developing seeds and/or presence of complex gene networks controlling the fluxes between the endoplasmic reticulum and the chloroplast within the peanut cells cannot be ruled out (Wang et al., 2015e).

## Conclusions

As per the US Food and Drug Administration determination, various health risks are associated with the consumption of trans-fat. As oleic acid is less prone to oxidation, the food products made from high oleic peanuts will have more shelf-life, which is most preferred trait of both oil and food processing industry. Such oil also has high impact on the markets due to the consumer's preference for several health benefits. Therefore, to make the oil healthier, breeding of improved peanut lines with high oleic and low linoleic acid becomes one of the most important breeding goals of recent times.

Though, India is one of the major oilseeds producing country, till now, there is no report of high oleate cultivar in any crop including peanut. Therefore, it is required to breed high oleate peanut cultivars in India, so as to enhance the livelihoods of small and marginal farmers, along with benefiting all the stakeholders of the value chain. Significant variation for oleic acid content was found in the genotypes that were identified having mutant *ahFAD2* gene; which confirms the complex mechanisms regulating O/L ratio, and involvement of more genes and other factors in increasing the linoleic acid. In this backdrop, phenotypic confirmation is essential to advance the selected lines for further evaluations.

The peanut genotypes identified having *ahFAD2A* mutant allele in homozygous condition, should be intensively used for the development of double mutant lines, using MAS. It is therefore proposed that the combined approach of both, genotypic- as well as phenotypic- based selection should be used for the effective identification of lines having mutant *ahFAD* alleles. Additional studies on the expression of other *FAD2* genes in various tissues may identify varying expression patterns of these genes including partitioning of expression. Moreover, further search for the novel mutant alleles or SNPs in *ahFAD* gene family are also underway in different laboratories of the world, which may help in significantly increasing increase the O/L ratio of different cultivars, in time to come. Currently, we are in the process of utilizing these assays using mutant lines for *ahFAD* alleles, in our peanut breeding programme to facilitate the development of lines with desired oil profile in a very precise and efficient manner.

## Author contributions

BN: Performed the experiments, analyzed the data and wrote the paper; TB: Analyzed the data; RT: Designed the experiments, wrote the paper; AR: Designed the experiments; AK: Performed the analysis; JD: Performed the experiments; RK: Designed the experiments; GM: Designed the experiment, performed analysis, wrote the paper.

### Conflict of interest statement

The authors declare that the research was conducted in the absence of any commercial or financial relationships that could be construed as a potential conflict of interest.

## Supplementary material

Figure S1 **Fatty acid profile of peanut genotypes**.

Figure S2 **Chromatograms of PCR sequencing showing heterozygous peaks at different locations**.

Figure S3 **Comparison of the deduced amino acid sequences of ahFAD2A gene in 30 peanut genotypes**.

Figure S4 **Cladogram generated from cluster analysis using ahFAD2B nucleotide sequence data of 30 genotypes**.

Figure S5 **Cladogram generated from cluster analysis using ahFAD2A and ahFAD2B combined nucleotide sequence data of 30 genotypes**.

Table S1 **Details of markers used for the identification of ***ahFAD2A*** and ***ahFAD2B*** mutant alleles in the peanut genotypes**.

Table S2 **List of peanut varieties selected for ***ahFAD2*** gene sequencing**.

Table S3 **Pedigree analysis of Indian cultivars for ***ahFAD2A*** mutant allele**.

Table S4 **Pedigree and fatty acid (Myristic, Linoleic and Erucic acid) composition (%), in the 167 peanut varieties and six advance breeding lines**.

Table S5 **Full length FAD2A gene sequence among selected peanut genotypes**.

Table S6 **Full length FAD2B gene sequence among selected peanut genotypes**.
