# Supplementary figures and images for: Insights into the Indian Peanut Genotypes for ahFAD2 Gene Polymorphism Regulating Its Oleic and Linoleic Acid Fluxes
Source: Front Plant Sci. 2016 Aug 25;7:1271. doi: 10.3389/fpls.2016.01271 (PMC4997015; doi:10.3389/fpls.2016.01271)

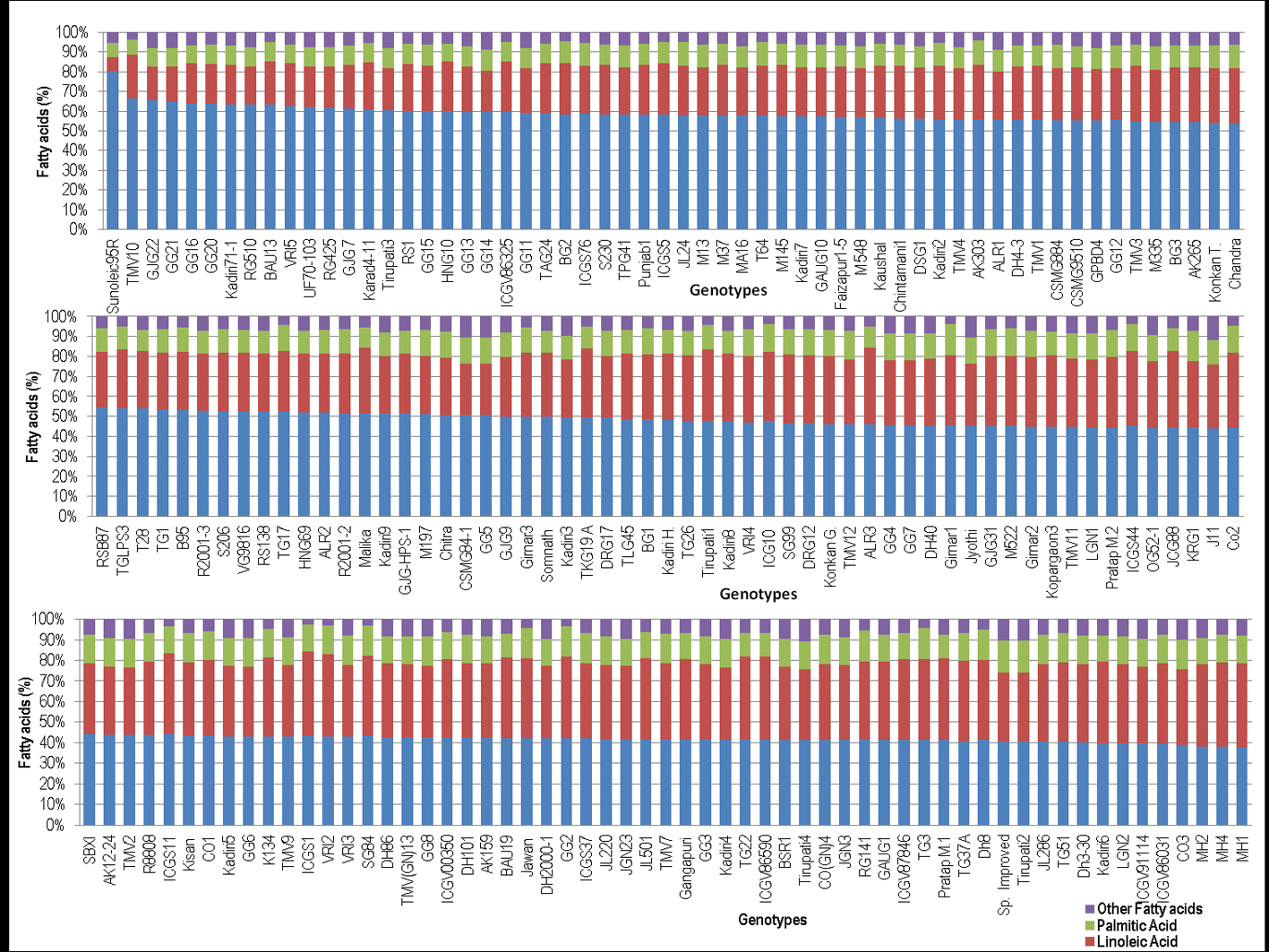

Supplement: Figure S1 — Fatty acid profile of peanut genotypes. [file Image1.TIF]

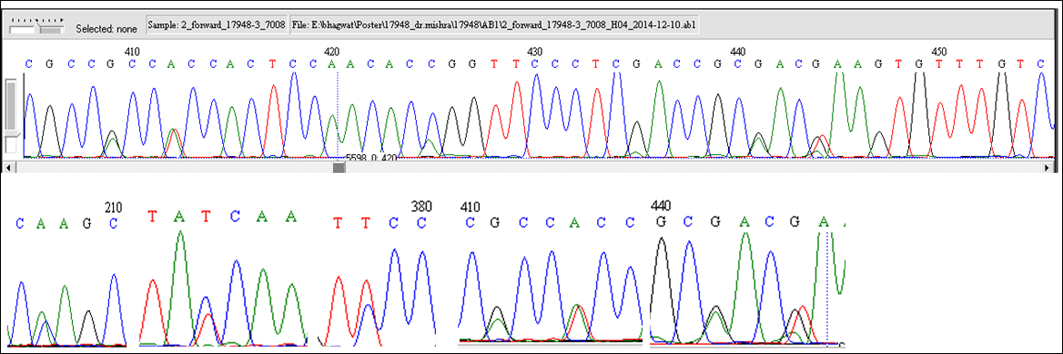

Supplement: Figure S2 — Chromatograms of PCR sequencing showing heterozygous peaks at different locations. [file Image2.TIF]

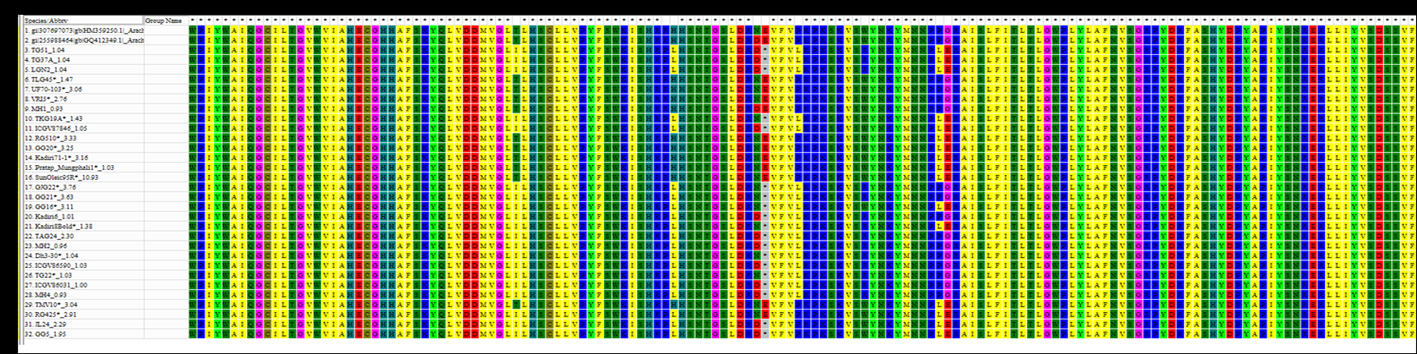

Supplement: Figure S3 — Comparison of the deduced amino acid sequences of ahFAD2A gene in 30 peanut genotypes. [file Image3.TIF]

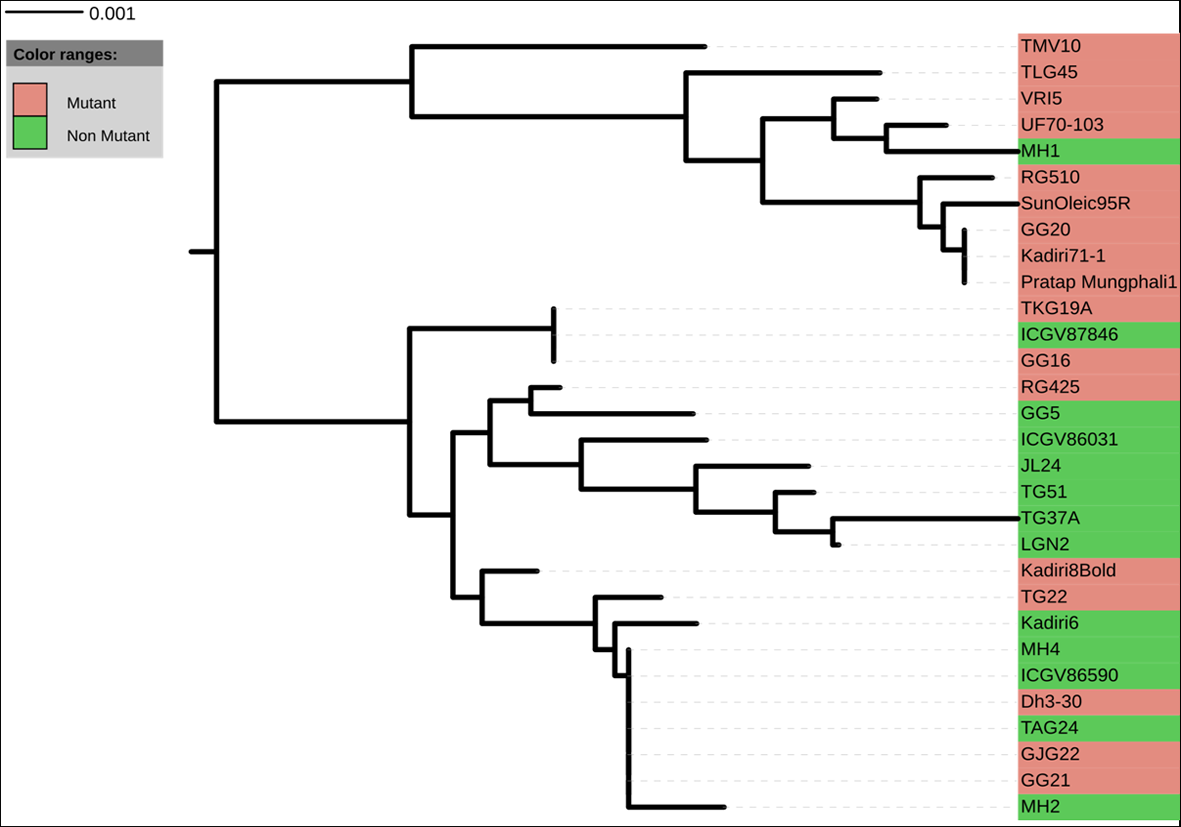

Supplement: Figure S4 — Cladogram generated from cluster analysis using ahFAD2B nucleotide sequence data of 30 genotypes. [file Image4.TIF]

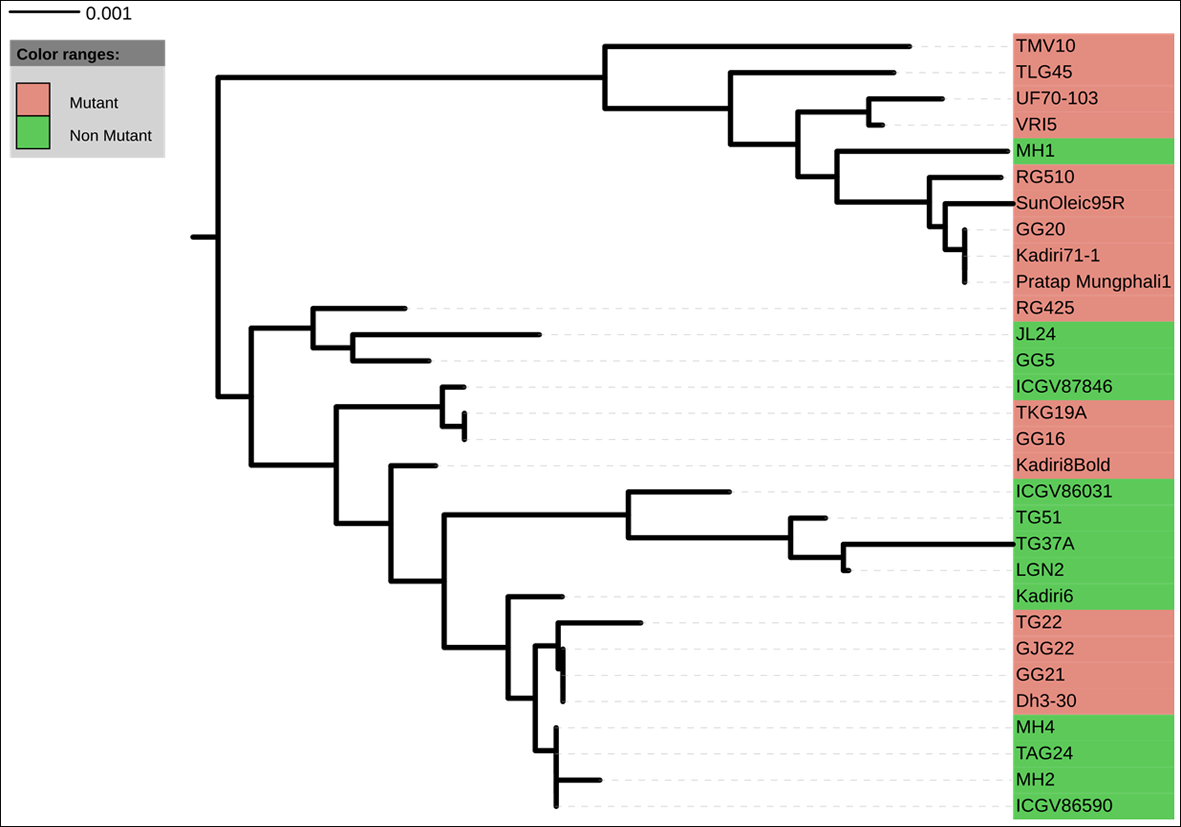

Supplement: Figure S5 — Cladogram generated from cluster analysis using ahFAD2A and ahFAD2B combined nucleotide sequence data of 30 genotypes. [file Image5.TIF]
